# T cell receptor clonotypes modulate the protective effect of human leukocyte antigen class I alleles in HIV-1 infection

**DOI:** 10.1186/1742-4690-9-S2-P264

**Published:** 2012-09-13

**Authors:** H Chen, ZM Ndhlovu, D Liu, LC Porter, JW Fang, S Darko, MA Brockman, T Miura, ZL Brumme, A Schneidewind, A Piechocka-Trocha, KT Cesa, J Sela, TD Cung, I Toth, F Pereyra, XG Yu, DC Douek, DE Kaufmann, TM Allen, BD Walker

**Affiliations:** 1 Ragon Institute of MGH, MIT and Harvard, Charlestown, MA, USA; 2 Vaccine Research Center, NIH, Chevy Chase, MD, USA

## Background

Human leukocyte antigen (HLA) class I alleles B*27 and B*57 are associated with protection against HIV-1 disease progression, but factors modulating the HLA protective effect remain unclear.

## Methods

Clonality of tetramer-sorted HIV-1 epitope-specific CD8 T cells from HIV-1 elite controllers and chronic progressors expressing HLA B*27 or B*57 were determined by T cell receptor (TCR) gene sequencing. Polyfunctionality, proliferation, avidity and differentiation phenotypes were analyzed by flow cytometry. Recognition of a GFP reporter cell line or autologous CD4 T cells infected with HIV-1 containing known mutations by HIV-1 epitope-specific bulk CD8 T cells or clonotypic CD8 T cell clones was analyzed by flow cytometry or analysis of p24 production. Immunological synapses between different CD8 T cell clones and infected target cells and lytic granule loading and delivery on a per cell basis were examined using three-dimensional confocal microscopy.

## Results

HLA-B*27-restricted CD8 T cells in controllers and progressors are quantitatively similar but clearly differentiate based on the ability to inhibit virus replication through targeting of the immunodominant Gag epitope. This in turn is associated with distinct TCR clonotypes, which are characterized by superior control of HIV-1 replication in vitro, greater cross-reactivity against epitope variants, and enhanced perforin expression and delivery at the immunological synapse. Clonotype-specific differences in antiviral efficacy were also observed for an immunodominant HLA-B*57 restricted response in controllers and progressors.

## Conclusion

These data indicate that the efficacy of protective alleles is modulated by specific TCR clonotypes that are selected in natural infection, and provide a functional explanation for divergent HIV-1 outcomes in persons with protective HLA alleles. Efforts to define the factors that contribute to junctional rearrangement of more effective TCR may be of critical importance for T cell vaccine design and therapeutic strategies for highly variable pathogens like HIV-1.

